# Maternal Venous Hemodynamic Dysfunction in Proteinuric Gestational Hypertension: Evidence and Implications

**DOI:** 10.3390/jcm8030335

**Published:** 2019-03-11

**Authors:** Wilfried Gyselaers

**Affiliations:** 1 Department of Obstetrics & Gynaecology, Ziekenhuis Oost-Limburg, Schiepse Bos 6, 3600 Genk, Belgium; wilfried.gyselaers@zol.be; Tel.: +32-89327524; 2 Department Physiology, Hasselt University, Agoralaan, 3590 Diepenbeek, Belgium

**Keywords:** gestational physiology, renal function, venous congestion, preeclampsia, gestational hypertension, small for gestational age, placentation, venous Doppler, maternal hemodynamics

## Abstract

This review summarizes current knowledge from experimental and clinical studies on renal function and venous hemodynamics in normal pregnancy, in gestational hypertension (GH) and in two types of preeclampsia: placental or early-onset preeclampsia (EPE) and maternal or late-onset (LPE) preeclampsia, presenting at <34 weeks and ≥34 weeks respectively. In addition, data from maternal venous Doppler studies are summarized, showing evidence for (1) the maternal circulation functioning closer to the upper limits of capacitance than in non-pregnant conditions, with intrinsic risks for volume overload, (2) abnormal venous Doppler measurements obtainable in preeclampsia, more pronounced in EPE than LPE, however not observed in GH, and (3) abnormal venous hemodynamic function installing gradually from first to third trimester within unique pathways of general circulatory deterioration in GH, EPE and LPE. These associations have important clinical implications in terms of screening, diagnosis, prevention and management of gestational hypertensive diseases. They invite for further hypothesis-driven research on the role of retrograde venous congestion in the etiology of preeclampsia-related organ dysfunctions and their absence in GH, and also challenge the generally accepted view of abnormal placentation as the primary cause of preeclampsia. The striking similarity between abnormal maternal venous Doppler flow patterns and those observed at the ductus venosus and other abdominal veins of the intra-uterine growth restricted fetus, also invites to explore the role of venous congestion in the intra-uterine programming of some adult diseases.

## 1. Introduction

Renal function during normal and pathologic pregnancy has been studied intensively, towards better understanding the clinically relevant sign of preeclampsia-related proteinuria. This research has mainly focused on pre- and intrarenal processes, whereas little attention has been given to aspects of renal venous function and outflow. Renal congestion following venous compression, (sub)obstruction or intravenous hypertension, is a well-known cause of renal dysfunction and even failure, both in experimental and in clinical conditions. Doppler studies of the maternal venous compartment have revealed important differences between normal pregnancies and those complicated with gestational hypertension (GH), early-onset (EPE) and late-onset preeclampsia (LPE). This review links the reported evidence from renal and venous physiology studies, relative to normal or abnormal course of pregnancy, bringing up new and challenging research questions on the role of venous hemodynamic dysfunction in the symptoms of preeclampsia-related organ dysfunction and on the pathophysiologic background mechanisms of preeclampsia.

## 2. Renal Physiology in Normal Pregnancy and in Two Types of Preeclampsia

Normal early gestational cardiovascular adaptation presents with overall vasodilatation and drop in systemic vascular resistance [1]. This is responsible for a relative intravascular underfilling, triggering volume retention mechanisms such as altered secretion of antidiuretic hormone, reset of threshold for thirst [2] and activation of the renin–aldosteron–angiotensin system [3]. Estrogen and other ovarian, decidual or placental products stimulate the release of various phenotypes of angiotensinogen (ATG), metabolized by renin from the renal macula densa to angiotensin I, which in turn is converted to the biologically active angiotensin II (AngII) via angiotensin convering enzyme (ACE). AngII is responsible for vasoconstriction, increased sensitivity for sympathic stimulation and release of aldosteron via the AT type 1 (AT1) receptor, and to a much lesser extent for vasodilatation, apoptose and reduced cell growth via the AT type 2 receptor [4]. Normotensive pregnant women are refractory to the vasoconstrictive effects of AngII due to AT1 inactivation by progesterone, prostacyclin and reactive oxygen species (ROS) [4].

In uncomplicated human pregnancy, intravascular volume rises 30% to 50%, and with this glomerular filtration rate increase with around 50% [5]. As a result, serum levels of creatinine, urea, uric acid, osmololality and sodium decrease, despite a net gain of around 1000 mg sodium without associated hypokaliemia. Morphologic kidney changes include increased renal diameters and volumes, and the majority of pregnant women show physiologic hydronephrosis, mostly right sided [5].

Preeclampsia is one of the most important complications of pregnancy, with major impact on maternal and neonatal morbidity and mortality [6]. It is characterized with new-onset hypertension beyond 20 weeks of gestation, associated with mild or severe signs of organ dysfunction, such as proteinuria, clotting disorders, liver and neural dysfunction, fetal growth restriction, … [7]. Renal function is compromised during preeclampsia, and glomerular endotheliosis is considered the histologic landmark of preeclampsia, characterized by endothelial swelling, loss of endothelial fenestrae with disruption of the glomerular filtration barrier and “empty” occluded capillary lumens [8]. These lesions are thought to result from glomerular endothelial dysfunction, probably mediated via placental solube fms-like tyrosine kinase-1 (sFlt-1) inactivation of podocyte-vascular endothelial growth factor (VGEF) and via soluble endoglin (sEng) inhibition of tranforming growth factor β, factors needed for a normal function of the glomerular endothelium [9]. Dysfunctional endothelium triggers further impairment of renal function via induction of podocyte dysfunction with subsequent podocyturia [10] and increased nephrin concentrations in serum and urine [11], but also via thrombotic micro-angiopathy [9]. The latter results from increased (afferent) arteriolar vascular and venular resistance [12] by inhibition of (a) endothelial derived gestational vasodilatation mediated via nitric oxide (NO)-dependent molecular mechanisms, (b) sympathetic sensitivity [13] and (c) relaxin [12]. Preeclampsia-related acute kidney injury results from ADAMTS-12 and 13 (a disintegrin and metalloproteinase with thrombospondin motifs) associated microangiopathy and from activation of the alternative and/or classical complement pathway [14,15]. Compared to normal pregnancy, glomerular filtration rate in preeclampsia is reduced despite maintenance of effective renal plasma flow (EFPR) and oncotic pressure [16], which suggests that structural glomerular damage is the main cause of preeclampsia-related proteinuria. Next to this, preeclampsia is also characterized with reduced proximal tubular reabsorption of intraluminal non-albumin proteins [17]. As such, the urinary content of around 50 different specific proteins is different in preeclampsia than in normal pregnancy, being the topic of many current studies on urinary proteomics [18], aiming for discrimination between two clinical phenotypes of preeclampsia: the early-onset and late-onset preeclampsia, depending on clinical presentation <34 weeks or ≥34 weeks respectively, also named the placental or maternal types of preeclampsia [19,20].

Renal handling of uric acid (UA) is altered in preeclampsia. In normal conditions, UA is filtered completely from the serum by the renal glomerulus, and nearly full reabsorption occurs at the S1 segment of the proximal tubulus. This is followed by UA intratubular secretion at the S2 segment [21]. The latter process is hampered in preeclampsia, resulting in increased UA serum concentrations. UA is an important mediator of endothelium function via inhibition of NO-release, stimulation of endothelin-1 production, enhancement of angiotensin II and smooth muscle contraction, with subsequent endovascular inflammation and c-reactive protein (CRP) release [21].

In preeclampsia, several phenotypes of AT1 are present, due to which angII sensitivity increases [22], despite of decreased circulating components of the renin–angiotension–aldosteron system (RAAS). This is explained by a more pronounced decrease of vasodilating than vasoconstricting circulating RAAS components [23]. Increase of aldosterone and plasma volume expansion is less pronounced in preeclampsia than in normal pregnancy [24,25] and this is associated with an increased interstitial concentration of salt with subsequent immune response [26]. Next to this, the number of placental AT1 receptors increases together with the alternative conversion to angII and subsequent increased release of endothelin 1. Women with preeclampsia show activity of an auto-antibody against the AT1 receptor, effecting the function of placenta, kidneys and other organs via (a) increased hypoxia induced SFlt-1 release interfering with angiogenesis, (b) increased placental and renal release of plasminogen activator inhibitor-1 (PAI-1) with reduced trophoblast invasion, reduced turn-over of extracellular matrix and increased renal sub-endothelial fibrin deposits, (c) stimulation of ROS production, (d) increase of intracellular calcium concentrations and (e) increase of coagulation by tissue factor (TF) [4,27]. Increase of AT1 auto-antibodies is more pronounced in LPE than in EPE [28]. On the other hand, homozygous angiotensin-converting-enzyme (ACE) genotypes were more frequent than in normal pregnancy in EPE but not in LPE [29].

During pregnancy, total body water (TBW) volume increases due to expansion of all maternal body fluid compartments, including intracellular, interstitial and functional extracellular volumes such as plasma volume, liquor, … [30]. Bio-impedance measurements have shown that overall TBW increase is more pronounced in preeclampsia than in normal pregnancy, and that this effect is more pronounced in the third trimester of LPE compared to EPE [31]. Next to this, it is well known that plasma volume (PV) expansion is less pronounced in preeclampsia than in uncomplicated pregnancy [32], however increased PV volume in LPE has also been reported [33,34]. Decreased PV expansion may result from constitutionally low plasma volume before conception, poor expansion due to dysfunctional mechanisms of neurohormonal volume retention [35] or increased leakage into the interstitium during the course of pregnancy [32]. The latter is supported by the well-known clinical presentation of manifest edema in pregnancy [30], associated with hemoconcentration [36] and with bio-impedance measured extracellular volume [31]. Another method to estimate an individual’s intravascular filling state non-invasively is the ultrasound derived inferior vena cava (IVC) collapsibility index (IVCI) [37]. IVCI is defined as (IVmax − IVmin)/IVmax, where IVmax is the IVC diameter at maximal inspiration and IVmin at maximal expiration. In patients at intensive care units (ICU), IVCI correlates well with invasively measured central venous pressure and pulmonary artery pressure [38,39]. In comparison to uncomplicated pregnancies, reduced IVCI was observed in LPE but not in EPE, suggesting a higher intravascular filling state in LPE than in EPE [40].

Vasopressin is synthesized and released together with co-peptine; compared to normal pregnancy, serum concentrations of co-peptine are higher in EPE but not LPE [41], and are already detectable before clinical onset of disease [42]. Similarly, serum concentrations of natriuretic peptides types A, B and C are higher in preeclampsia than in normal pregnancy, which is particularly true for EPE as compared to LPE [43,44,45,46,47].

## 3. Abnormal Venous Hemodynamics and Renal Dysfunction in Experimental Conditions and Clinical Syndromes

Dysfunction of the venous compartment affects organ function, both in experimental conditions [48,49,50,51,52,53] and in clinical syndromes such as renal or hepatic vein obstruction/thrombosis, cirrhotic cardiomyopathy and the cardiorenal syndrome [54,55,56,57,58,59]. Impaired organ function results from (a) increased venous pressure causing microcirculatory congestion, and (b) compromised organ perfusion due to arteriolar constriction, secondary to raised sympathetic activity. The concept of renal dysfunction as a result of venous congestion being transmitted to the renal veins and kidneys has been supported by a wide range of studies since the 1930s. In an experimental model with induced hypervolemia, an increase in renal vein pressure caused renal insufficiency, irrespective of cardiac output and renal blood flow [60,61]. Other studies indicated that transient renal vein compression reduced sodium excretion, glomerular filtration rate (GFR) and renal blood flow [62,63,64]. Also, an increase in central venous pressure has been found to increase renal interstitial pressure, most likely leading to renal hypoxia, resembling congestion and dysfunction of the liver as observed during cardiac failure [65,66,67,68,69,70,71]. In addition to these mechanically induced effects, an increase in venous pressure can activate RAAS [65,72]. The latter will further reduce ERPF and GFR [73,74,75,76,77,78]. Another sequel to elevated central venous pressure is the development of visceral edema and ascites, both likely to aggravate dysfunction of the intra-abdominal organs and kidneys [79,80,81]. Venous congestion rather than impaired cardiac output has been shown to be the most important trigger for deteriorating renal function in patients with advanced low-output heart failure [59,82,83]. In this context it is important to emphasize that renal dysfunction due to venous congestion almost always reverses rapidly after lowering the renal venous pressure or the intra-abdominal pressure as indicated by an immediate recovery of diuresis and GFR [60,61,79,82,84].

## 4. Venous Adaptations in Normal Pregnancy and in Early- or Late-Onset Preeclampsia

Already from the very early stages of pregnancy, the venous system is involved in the maternal cardiovascular adaptation process: human trophoblast cells not only invade spiral arteries, but also lymphatics and veins to create an open communication canal with the intervillous space [85,86]. The invasion of decidual veins already occurs before spiral artery remodeling [87], affects a larger fraction of veins than arteries [88], is associated with intravenous fibrin deposition [89] and with venous dilatation [90]. Reduced extravillous trophoblast invasion of venous and lymphatic vessels with unaltered spiral artery remodeling has been linked with recurrent miscarriage [88].

Experimental rat models showed that pregnancy not only induces a coordinated and multifaceted remodeling of the uterine veins, but also a dilatation of mesenterial veins with subsequent increased capacitance at the cost of compliance [91]. The latter mechanism seems endothelium dependent [92]. As such, the capacitance function of the splanchnic bed is increased in pregnant women and serves the meticulous control of cardiac output, supporting maintenance of constant uterine flow volume under most physiologic conditions. This is supported by the reported correlations between hepatic venous Doppler flow, cardiac output and neonatal birthweight [93].

One method for non-invasive assessment of venous hemodynamic function in pregnant women is venous Doppler sonography. Doppler assessments at the venous compartment are much more complex than at the arterial site, not only due to technical and human limitations [94], but also as a result of the low venous flow velocities and pressures, the interferences by multiple physiologic variables and the large anatomic variations of the venous compartment [95]. With sonographer’s training and the use of repeated measures, an electrocardiography- (ECG) assisted venous Doppler sonography technique allows achieving intra- and interobserver correlation coefficients >0.9 [96].

**Figure 1:**
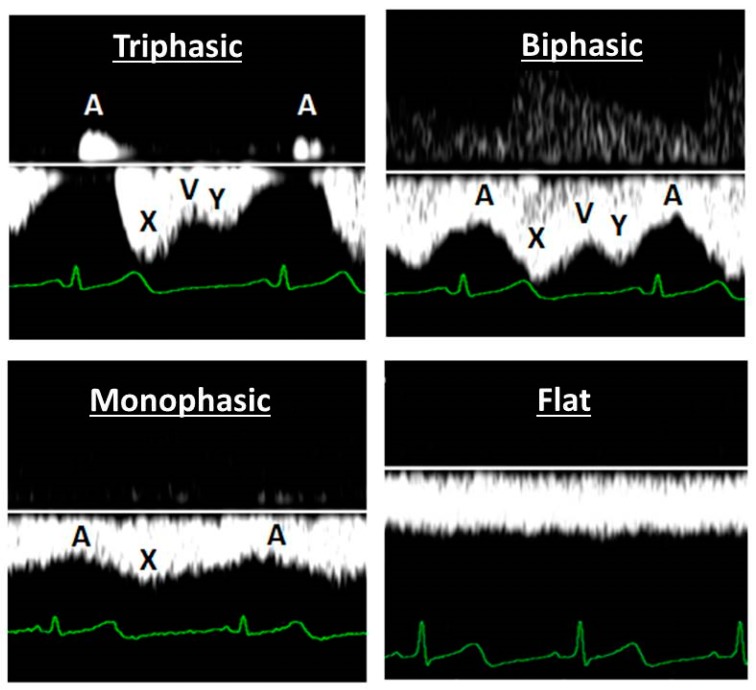
Four common types of venous wave forms observed during Doppler sonography: triphasic, biphasic, monophasic and flat. The A-deflection represents retrograde flow of venous blood from the heart into the vena cava during atrial contraction, in a close time relationship with the electrocardiography P-wave. The X- and Y-deflections represent forward venous flow caused by atrial and ventricular diastole respectively. V represents the opening of the tricuspid valve.

Venous Doppler wave characteristics are those of the well-known jugular vein pulse, and reflect the cyclic changes at the right heart [97]. Negative suction forces during diastolic relaxation of the right atrium and ventricle are responsible for forward venous flow, reflected in the X- and Y-wave respectively (Figure 1). Due to lack of a valve mechanism at the right atrial inflow, atrial systole is responsible for a retrograde jet of venous blood into the vena cava, which in non-pregnant individuals reflects as a reversed A-wave at the level of the hepatic veins, or as a deceleration of forward flow at the level of renal interlobar veins [98] (Figure 1).

Venous Doppler flow characteristics are measured quantitatively using the so-called venous impedance index (VI) [99] and/or venous pulse transit (VPT) [100]. VI is the venous Doppler equivalent of arterial resistance index (RI), defined as (max velocity − min velocity)/max velocity. Its value is considered to relate to intravenous pressure [99], and depends on intravenous volume, venous vascular tone and transmural and/or intra-parenchymatous pressure [101]. Under experimental conditions, intravenous volume load results in higher A-wave peak velocity, whereas valsalva manoevre is responsible for flattening of the Doppler wave form [102]. VPT is measured as the heart rate corrected time interval between the ECG P-wave, initiating atrial contraction, and the Doppler venous A wave, resulting from atrial contraction [100]. The magnitude of this ratio depends on vascular tone and the distance between the point of measurement and the heart [103]. As such, constrictive venous vascular wall activity results in high VI and low VPT, whereas passive venous vascular wall elasticity presents with low VI and high VPT.

In non-pregnant conditions, venous flow velocities are higher in right than in left kidney, as a result of side-specific anatomical differences [104,105]. Longitudinal observations have shown a sinusoidal pattern of renal interlobar VI with a slow frequency of 10–14 days, and with an alternating pattern in left and right kidney [106].

**Figure 2:**
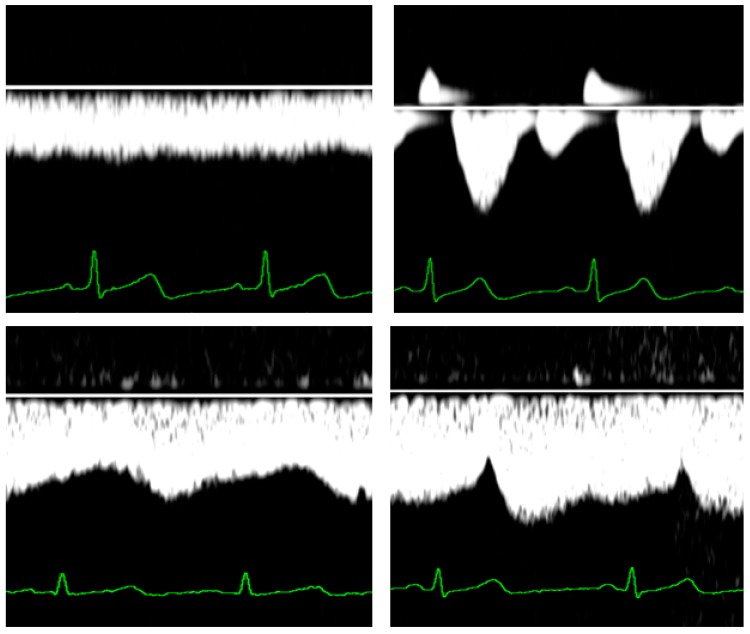
Venous Doppler wave forms at the level of the liver (upper panels) and the kidneys (lower panels) during normal third trimester pregnancy (left panels) and during early onset preeclampsia (right panels). The latter condition is specifically associated with the so-called venous pre-acceleration nadir (VPAN), illustrated in the right lower panel.

Important changes of venous Doppler characteristics during uncomplicated pregnancy were reported at the level of maternal liver [107] and kidneys [108]. This change is most pronounced at the level of hepatic veins, where triphasic patterns in the first trimester become totally flat near term [109] (Figure 2). At the level of renal interlobar veins, this change is less dramatic from biphasic to monophasic patterns (Figure 2).

Both maximum and minimum renal interlobar vein flow velocities increase during the first trimester, plateau during the second and show a slight decrease near term [105]. As a result, VI decreases in both left and right kidney, in concert with increase of venous pulse transit [100]. These changes are consistent with reported increase of venous distensibility in pregnancy [110] and with increased renal perfusion and venous outflow [5]. In the third trimester, right kidney RIVI is lower than in the left kidney [105]. There is also a reduction of sinusoidal undulation of VI as compared to non-pregnant conditions, with persistently lower values in the right than in the left kidney [106].

Venous Doppler flow changes in uncomplicated pregnancies differ from those observed during preeclampsia [99]. During the clinical stage of EPE, retrograde atrial contraction rebound via the venous system is observed up to the level of the kidneys in the so-called venous pre-acceleration nadir (VPAN) [98]. This observation suggests an intermittent pulsed counterforce against renal venous drainage at every heartbeat, an under-recognised pathophysiologic mechanism potentially contributing to progressive renal dysfuction (Figure 2).

**Figure 3:**
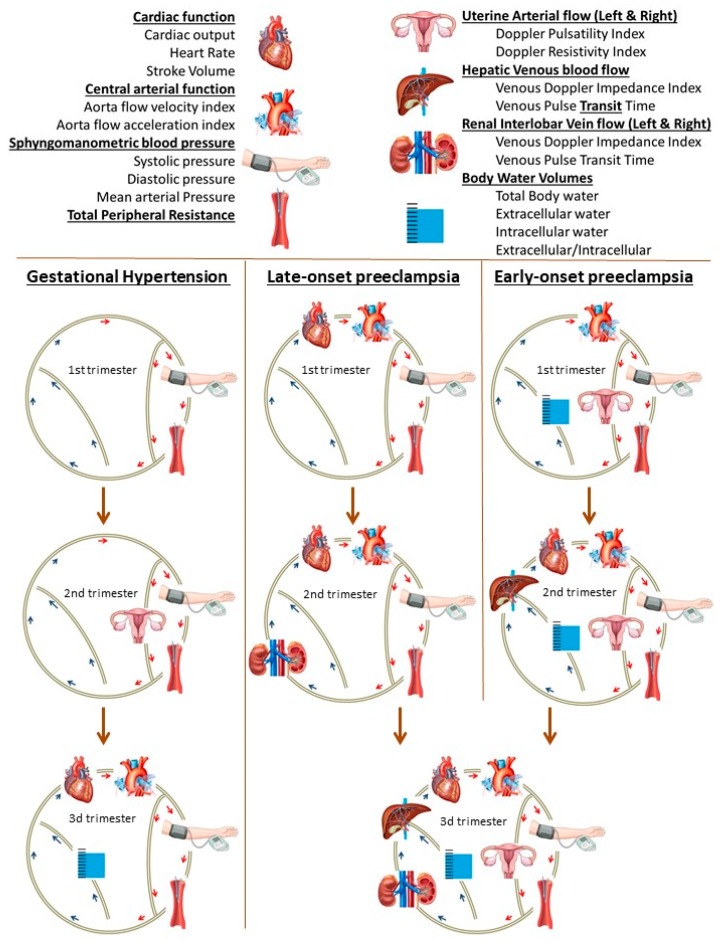
Graphical presentation of the gradual deterioration of the maternal circulation from first to third trimester in pregnancies, destined to develop gestational hypertension, early- or late-onset preeclampsia [113]. The maternal cardiovascular system is depicted as a circle, with different components and parameters, explained in the legend on top. Normal functioning components are not shown in the diagram; the depicted components are those where abnormal measurements were obtained, relative to normal pregnancies. As is shown, each gestational hypertensive disorder presents in the first trimester with a unique combination of dysfunctional cardiovascular components, and with advancing pregnancy this is followed by a type-specific pathophysiologic pathway. Characteristics of abnormal venous hemodynamics are present in early- and late-onset preeclampsia, but not in gestational hypertension.

Another very important observation with intrarenal venous Doppler sonography is that abnormal measurements of both VI and VPT have been found in women with preeclampsia, but not in women with gestational hypertension [111,112,113]. These abnormalities are more pronounced in EPE than in LPE [114]. As compared to uncomplicated pregnancies, abnormal VI in EPE is observed weeks before clinical onset of proteinuria [115], presents with a more pronounced sinusoidal undulation with parallel course in both kidneys [106]. In LPE, abnormal VI is lower than in EPE, can present unilaterally and usually is observed from onset of proteinuria [114,115]. A weak but significant correlation is reported between VI and degree of proteinuria in LPE but not EPE [116]. Abnormal VPT is also limited to preeclampsia as it is not altered in gestational hypertension [111]. Similar to VI, VPT is more abnormal in EPE than in LPE [112]. The time onset and type of venous hemodynamic dysfunction during the course of pregnancy is also different between EPE and LPE [115,117]. In EPE, hepatic vein VI can be abnormal from the second trimester onward and presents before abnormal VI of renal interlobar veins, whereas this is opposite for LPE [113]. This is illustrated in Figure 3.

Another important observation related to renal function in pregnancy, is that all pregnancies, regardless of maternal or fetal outcome, are subject to an expansion of body water volume. Features of abnormal volume homeostasis already are present from the first trimester onward in EPE and normotensive poor fetal growth (defined as birthweight less than 10th percentile = small for gestational age), and from the second trimester onward in LPE and GH [31].

## 5. Increased Risk for Chronic Renal Disease and/or Persistent Hemodynamic Dysfunction after Gestational Hypertension and Preeclampsia

Incidence of hypertensive disorders of pregnancy vary between countries, ranging between 1.4%–4.0% for preeclampsia overall, 0.3%–0.7% for early onset preeclampsia and 3.6%–9.1% for gestational hypertension [118].

Formerly preeclamptic women are at increased risk for development of long term cardiovascular [119,120] and renal disease [121,122]. This association is more pronounced in early-onset than in late-onset preeclampsia [123,124], the highest risk is in chronic hypertensive women with superimposed preeclampsia [125] and is independent of endothelium dysfunction [126]. This association has been linked to presence of subclinical and undiagnosed renal disease [127,128].

Similarly, chronic renal disease also develops more frequently in women with a history of non-proteinuric gestation-induced hypertension. Also in this population, undiagnosed subclinical renal disease is common, as is confirmed by postpartum renal biopsy [129] and by higher recurrence in women with suboptimal rise of glomerular filtration in early subsequent pregnancy [130]. One possible explanation is that these women gave birth before the gestational hypertensive disease had reached the full clinical stage of preeclampsia: non-proteinuric hypertension has been observed to present weeks before onset of proteinuria [114,131], and non-proteinuric types of preeclampsia have been reported [132] as well as interfering co-morbidities such as insulin-resistance [133].

A large fraction of women with a history of preeclampsia also show low plasma volume, predisposing to increased recurrence risk [134]. This is particularly true for women with early gestational cardiac dysfunction in the subsequent pregnancy [135,136]. Low plasma volume is associated with signs of reduced venous compliance and baroreceptor sensitivity, and with increased sympathetic tone [137]. An integrated assessment of plasma volume, venous hemodynamics and renal function in short and long term postpartum of women with different types of gestational hypertensive disease has not yet been reported.

## 6. Implications for Clinical Practice

The role of maternal venous hemodynamic function in normal pregnancies and in gestational hypertensive disease, as outlined above, has important clinical implications. Firstly, it should be appreciated that the venous system is not a passive but a very active physiologic component of the circulation, with different properties than the arterial system, however equally important. In pregnancy, this is illustrated by the reported correlations between hepatic venous flow, maternal cardiac output and neonatal birthweight percentile [94]. Despite the technical difficulties in evaluating venous hemodynamics, the large anatomo-physiologic variations and the multiple interfering factors, an assessment of the cardiovascular circulation as a closed circuit can no longer be considered complete without any information on the veins in collaboration with the other components [114].

Secondly, the assessment of maternal venous hemodynamics opens perspectives towards improved screening for or diagnosis of gestational hypertensive diseases. While a lack of venous hemodynamic dysfunction relative to normal pregnancy is observed in gestational hypertension [112,113], the time onset, pattern, laterality, and sequence of venous Doppler abnormalities clearly differ between early and late onset preeclampsia. This allows implementing maternal venous Doppler sonography in the diagnostic work-up of women presenting with hypertension in pregnancy. Similarly, the presentation of abnormal venous Doppler parameters weeks before clinical onset of early onset preeclampsia opens perspectives towards implementation into screening algorithms for preeclampsia [116]. However, for this, the large interindividual variation in time onset of venous dysfunction still requires a longitudinal series of assessments [138].

Thirdly, the venous compartment is an important target for interventions before or during pregnancy, as is observed in beneficial effects of physical exercise [139,140], and also for pharmacologic treatment of preeclampsia [141]. From this perspective, three types of drugs are particularly of interest in the management of gestational hypertensive diseases: (a) magnesium (b) Nitric oxide (NO-)donors, and (c) diuretics.

Magnesium sulphate has been used for a variety of obstetrical indications, such as tocolysis, prevention and treatment of eclampsia and neonatal neuroprotection [142]. Despite its widespread application, the mechanisms of action are poorly understood [143]. A role for magnesium has been reported in the physiologic control of blood pressure and the pathophysiology of hypertension [144]. Reversal of vasospasms offers potential to magnesium supplementation as a pharmacologic treatment for cerebral or coronary vasoconstriction [145,146], an effect considered to be mediated via the NO-dependent endothelium pathway [147]. Experimental evidence from laboratory models and animal studies support magnesium-induced vasorelaxation activity in both the arterial and venous compartment, with maximal dilation of the venous capacitance vessels even at relatively low concentrations [148].

Nitroglycerin and other nitrates are well known endothelium dependent venodilating agents [149], with successful application in the management of preeclampsia, eclampsia and HELLP syndrome (hemolysis, elevated liver enzymes, low platelets), with or without pulmonary edema [150,151]. Improvements of abnormal Doppler flow measurements in uterine and umbilical arteries during nitroglycerin administration have been reported [152,153]. More recently, NO-donors have come into attention of obstetric researchers again, mainly because of the combination of beneficial cardiovascular effects with maternal and fetal safety [154]. NO-donors associated with plasma volume expansion have shown to improve diastolic blood flow velocity in the umbilical artery, in parallel with a reduction of maternal peripheral arterial resistance [155], both with beneficial effects on maternal and neonatal outcome [156].

Despite the use of diuretics as antihypertensive agents in postpartum of women with preeclampsia [157], abstinence from application during pregnancy has long been advocated because of the observed increase of peripheral resistance in a group of pregnant women with chronic hypertension [158]. The lack of terotogenic or clinical neonatal side effects in pregnancies with maintenance of chronic diuretic treatment or with acute cardiac or nephrologic problems [159,160], has stimulated the National High Blood Pressure Education Program Working Group on High Blood Pressure in Pregnancy to formulate the statement that the concern for the use of diuretics in pregnancy should be considered primarily theoretical [161,162]. These arguments, together with the recognition of two types of preeclampsia, has initiated research into the value of diuretics in the management of late onset preeclampsia, with preliminary promising effects [163].

Before the concept of the maternal venous compartment as a target for treatment of gestational hypertensive disease can be introduced into clinical practice, more experimental, clinical and epidemiological research is required.

## 7. New Hypothesis-Driven Research on the Role of Maternal Venous Hemodynamic Dysfunction in Preeclampsia

From the evidence outlined above, it is concluded that venous hemodynamic function during normal pregnancy differs from non-pregnant conditions in 3 fundamental aspects: (a) venous vascular tone is reduced, due to which venous capacitance function is increased, (b) the venous compartment participates in the gestational body water volume expansion, contributing to maintenance of optimal cardiac output under different physiologic conditions (c) transmural venous pressure is increased as a result of high intra-abdominal pressure due to the growing uterus, which is responsible for a rise of intravenous pressure. The net result of this is that the venous system functions closer to its maximum limits during pregnancy than in non-pregnant condition and as a result, the global cardiovascular system of women in advanced pregnancy functions close to the edge of “overload”. This is clinically visible in the presentation of malleolar edema in pregnant women near term [30]: a high-volume load in a venous compartment at maximum capacitance is responsible for capillary dysfunction in retrograde direction and increased extravasation of plasma constituents. This view is also supported by the large proportion of healthy pregnant women showing signs of cardiac volume overload at term [164], and also in the large variation of hepatic vein impedance measurements during the normal third trimester [110]. The latter reflects a high activity of liver hemodynamics in late pregnancy, contributing to the correlation between maternal cardiac output and neonatal birthweight [94].

Another important topic for future research is the observation of venous hemodynamic dysfunction present in women with preeclampsia but absent in women with gestational hypertension [112,113]. The clinical difference between PE and GH is that the latter does not present with symptoms of organ dysfunction, such as renal proteinuria, liver dysfunction, clotting disorders, convulsions… [7]. As such, the question arises whether the symptoms of PE-related organ dysfunction are triggered via the pathway of venous congestion, a phenomenon well known in the pathophysiology of diastolic heart failure [60,83] and some subtypes of cardiorenal [165] and cardiohepatic syndrome [166]. In humans, preeclampsia related microcirculatory dysfunction has been linked to the endothelium [167], and is associated with endothelial abnormal retrograde transmission of vasodilatory signals with subsequent precapillary flow reduction or even stop [168]. From this perspective, the observation of secondary hypertension in pregnant ewes after ligation of the uterine vein is very interesting [53] because of the important implication that arterial hypertension can occur as a consequence of abnormal venous hemodynamic function.

In the pathophysiologic process of venous congestion, an important contribution by intra-abdominal pressure (IAP) has been reported [80,82]. IAP is reported to increase well above normal values during pregnancy [169]. From this perspective, it is hypothesized that venous congestion in preeclampsia can occur via 3 different pathways, alone or in combination: (a) venous hypertension, associated with increased venous vascular tone, (b) venous overfill in a system functioning at maximum capacitance, and (c) increased external venous pressure as in intra-abdominal hypertension syndrome. Each of these mechanisms can trigger a cascade of cardiovascular humoral, paracrine of neural reflex responses with subsequent hypertension and reduced organ perfusion [170,171]. Interestingly, from the clinical point of view, these three mechanisms go very well with the reported subtypes of preeclampsia and their intrinsic longitudinal hemodynamic changes: (a) EPE with a sudden onset and fulminant course, often ending in a premature birth of an neonate small for gestational age [172,173], (b) one of two variants of LPE [174], in which a cross over occurs from an early gestational high volume/low resistance to a late gestational low volume/high resistance circulation [175], and (c) the second variant of LPE, where a high volume/low resistance circulation persists throughout the course of pregnancy in a population of mainly obese women [176]. Increased first trimester serum markers of endothelial activation together with increased Doppler pulsatility index and “notching” of the uterine arteries, can link EPE to a pathophysiologic state of overall vascular hypertonia. Endothelial activation by intravascular volume overload, as is seen during diastolic heart failure, can explain the process of cross over from a high volume/low resistance to a low volume/high resistance circulation in the first variant of LPE. Venous congestion secondary to external venous compression in a state of high intra-abdominal pressure, can explain the second variant of LPE.

From the discussion outlined above, another fundamental aspect for future research pops up: what is the role of the venous system in the earliest stages of embryo implantation and placentation? As explained, human trophoblast invasion is not limited to spiral arteries only but occurs earlier and more pronounced in lymphatics and veins [86,87,88,89,90,91]. It is most likely that Doppler sonography is an inadequate technology with poor sensitivity for detection of very subtle dysfunctions of the venous compartment in these very early stages of pregnancy. From the theoretical point of view however, it can be hypothesized that an implanting conceptus, who is confronted with an ineffective venous drainage from the uterus, uses incomplete remodeling of the spiral arteries as a protective mechanism against increased pressure [177,178] or congestion [179] in the intervillous space. This atypical remodeling of the uterine and placental circulation is reported without any associated process of oxidative stress, a concept that is generally accepted today on theoretical grounds only [180]. The hypothesis of a hemodynamic mechanism, underlying inadequate remodeling of the uterine vasculature, is supported by the clinical observation that pregnant women with congenital heart disease are at risk for preeclampsia, particularly when right heart dysfunction is involved [181].

A final area for future research comes up from the striking resemblance between types of Doppler wave forms in maternal and fetal abdominal veins [182], and between abnormal maternal hepatic venous Doppler patterns in EPE and the abnormal Doppler wave form at the level of the ductus venosus in the intra-uterine growth restricted fetus (IUGR) [183]. Abnormal fetal venous Doppler wave forms present in association with abnormal trans-tricuspid flow patterns, suggestive for fetal right heart diastolic dysfunction [184]. Maternal diastolic cardiac dysfunction is a well-known feature during preeclampsia, being more pronounced in EPE than LPE [45]. Without any doubt, the interpretation of the ductus venous Doppler flow deflections is similar to that of the triphasic pattern at the level maternal hepatic veins (Figure 1) and in the jugular vein [98], representing phases of the cardiac cycle in the right atrium. In accordance with the pathophysiologic interpretation of abnormal maternal venous Doppler flow patterns explained above, a triphasic ductus venosus Doppler waveform suggests for the IUGR fetus a state of activated venous hemodynamics, possibly intravenous hypertension predisposing to congestion related organ dysfunction. As fetal urine production is an important contributor to the amniotic fluid volume [185] and composition [186], the question arises whether renal venous congestion is an underrecognized mechanism underlying the well-known condition of oligo-amnion in IUGR. In parallel, fetal renal venous congestion may also contribute to the link between the reduced number of nephrons in IUGR neonates as compared to those with normal birthweight [187], and the related predisposition for early onset end stage renal disease [187,188,189], cardiovascular disease [190] and dysfunctions of other organ systems [191] in adults who used to be dysmature newborns. Understanding the pathophysiologic background mechanisms behind the intra-uterine programming of adult disease is the first step towards prevention, timely detection and targeted follow up of individuals at increased risk.

## 8. Conclusions

Maternal cardiovascular adaptations during pregnancy are associated with major changes of renal function. Similarly, abnormal circulatory function in complicated pregnancy, such as in early- or late-onset preeclampsia, co-exists with renal dysfunction but also with maladapted venous hemodynamic function. From the pathophysiologic point of view outlined in this paper, this hemodynamic dysfunction may have a much more important role to play in the etiology of preeclampsia-related symptoms of organ failure and as a target for clinical diagnosis and therapy, as currently considered today. More research is needed, both experimental and clinical, to explore further the role of renal venous hemodynamics in normal and abnormal renal function during pregnancy and preeclampsia, in the early gestational process of uterine vasculature remodeling, and in the intra-uterine programming of adult disease.
